# Is what you see what you get? The relationship between field observed and laboratory observed aphid parasitism rates in canola fields

**DOI:** 10.1002/ps.7002

**Published:** 2022-06-07

**Authors:** Samantha Elizabeth Ward, Paul A. Umina, Hazel Parry, Amber Balfour‐Cunningham, Xuan Cheng, Thomas Heddle, Joanne C. Holloway, Caitlin Langley, Dustin Severtson, Maarten Van Helden, Ary A. Hoffmann

**Affiliations:** ^1^ School of BioSciences, Bio21 Institute The University of Melbourne Parkville Victoria Australia; ^2^ Cesar Australia Level 1, 95 Albert Street Brunswick Victoria Australia; ^3^ CSIRO Entomology, Black Mountain Canberra ACT Australia; ^4^ Department of Primary Industries and Regional Development 75 York Road Northam Western Australia Australia; ^5^ South Australian Research and Development Institute, Entomology Urrbrae South Australia Australia; ^6^ New South Wales Department of Primary Industries, Wagga Wagga Agricultural Institute Wagga Wagga New South Wales Australia; ^7^ The University of Adelaide Adelaide South Australia Australia

**Keywords:** Aphididae, Aphidiinae, biological control, hymenoptera

## Abstract

**Background:**

Estimating parasitoid abundance in the field can be difficult, even more so when attempting to quantify parasitism rates and the ecosystem service of biological control that parasitoids can provide. To understand how ‘field observed’ parasitism rates (in‐field mummy counts) of the green peach aphid, *Myzus persicae* (Sulzer) (Hemiptera: Aphididae) translate to ‘laboratory observed’ parasitism rates (laboratory‐reared parasitoid counts), field work was undertaken in Australian canola fields, over the winter growing season.

**Results:**

Overall, laboratory observed parasitism was on average 2.4 times higher than field observed parasitism, with rates an average of four‐fold higher in fields from South Australia. Total field observed and laboratory observed parasitism rates (OPRs) of *M. persicae* varied considerably across regions, but less so among fields within regions. As crop growth stage progressed, the incidence of field observed mummies increased. The incidence of total parasitoids reared also increased with crop growth stage, averaging 3.4% during flowering and reaching 14.4% during podding/senescing. Although there was a greater diversity of reared parasitoid species at later crop growth stages, the laboratory OPR was unaffected by parasitoid species. *Diaeretiella rapae* was the most commonly reared parasitoid, increasing in absolute abundance with crop growth stage.

**Conclusion:**

These findings indicate that field mummy counts alone do not provide a clear representation of parasitism within canola fields. © 2022 The Authors. *Pest Management Science* published by John Wiley & Sons Ltd on behalf of Society of Chemical Industry.

## Graphical abstract

Total field observed and laboratory observed parasitism rates of *Myzus persicae* varied considerably, indicating that mummy counts alone do not provide a clear representation of aphid parasitism in canola fields.

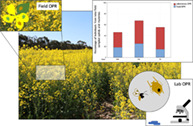

## INTRODUCTION

Estimating parasitoid activity in the field can be difficult, even more so when attempting to understand in‐field parasitism rates.^1^ Parasitoids protect themselves by pupating within the eaten‐out husk of their aphid host after cementing it to a substrate. This golden yellow (or brown/black) husk is referred to as a ‘mummy’,^2^ and at this stage a parasitized aphid can be identified visually. Growers and agronomists may use in‐field mummy counts as an indicator of aphid parasitoid activity. These findings inform their pest management decisions. If there are sufficient numbers of aphid mummies, and hence parasitoids, there may be less need to use chemical sprays to control pest aphid populations. However, these crude counts using mummies can prove inaccurate.^3^

Numerous biotic and abiotic factors can affect the development of mummies, causing further imprecision in the assessment of parasitoid wasp presence from in‐field mummy counts. The mummified husk of parasitized aphids forms approximately 72 h after oviposition at 23 °C.^4^ Based on visual monitoring alone, within this window, any aphid mummified at this temperature in the field could be incorrectly presumed to be unparasitized. Uncertainty is likely to be higher as temperatures decline and/or become more variable. Effect of temperature can vary depending on the parasitoids’ developmental stage. Temperature thresholds for aphidiines can be lower during the egg‐mummy stage than the mummy‐adult stage and vary across species.^5^ In addition, heat waves (i.e., extended periods of abnormally hot temperatures) can increase developmental time of aphid parasitoids.^6^ Other weather events such as strong wind or heavy rain can also dislodge mummies from plant leaves,^7^ reducing observed parasitism rates (OPRs).

Ecological factors such as intraguild predation, aphid health, and presence of endosymbionts can further reduce the success of parasitoid development.^8^, ^9^ The presence of plant virus can cause aphid hosts to become viruliferous. This results in higher mortality and longer developmental times of parasitoid larvae, and thus lower percentages of mummification.^10^ Facultative endosymbionts can decrease parasitoid survival in the aphid host.^11^ Additionally, the presence of other aphid species can affect host choice of parasitoids, which may prefer particular species and/or aphid instars.^12^, ^13^ Farming inputs will also affect parasitoid wasp populations. The application of nitrogen fertilizer can increase the percent emergence of parasitoids,^14^ while pesticide usage can reduce parasitoid emergence.^15^, ^16^ Even ecological pesticides, such as neem [*Azadirachta indica* (A. Juss.)] seed oil (NSO), can have a deleterious effect on parasitoid emergence.^17^

In the field, regardless of host species, many parasitoids are likely to die during development, with Nielsen and Hajek^18^ noting an overall rate of 56% non‐emergence across hosts. Dissection of mummies from which no parasitoid emerged revealed the parasitoid to be either dead or diapausing.^19^ Walton et al.^19^ found that mummy counts generally underestimated parasitism levels when compared with live rearing or DNA‐based techniques. Giles et al.^20^ also noted a weak relationship between the proportion of tillers with mummies against the proportion of parasitized aphids in wheat. These authors suggested that simultaneous aphid and parasitoid sampling is required instead to determine the value of applying pesticides to control pest aphids.^20^ Powell et al.^1^ also suggested that aphid mummy counts should be combined with other methods to estimate parasitoid abundance, because the sampling method alone could produce misleading results. These findings reinforce the notion that mummy counts alone are unlikely to provide sufficient information to accurately estimate parasitoid activity.

The green peach aphid, *Myzus persicae* (Sulzer) (Hemiptera: Aphididae), is an important agricultural pest worldwide and was first recorded in Australia in New South Wales (NSW), in 1910.^21^ Within Australia, in the cooler regions, *M. persicae* can overwinter as eggs on peach (*Prunus persica* L.), its primary host, with female apterae hatching in early spring onwards.^22^ Later, female alates are produced, dispersing onto host plants to reproduce over summer and autumn.^22^ In other regions, *M. persicae* is anholocyclic, with females giving birth to live young that are genetic clones of their mother. Combined with a short generation time, this mode of reproduction allows *M. persicae* populations to increase rapidly under favorable conditions. A study in Australia showed *M. persicae* to be the most abundant aphid species in canola fields from southern and western grain production areas.^21^ *Myzus persicae* populations can fluctuate from year to year,^23^ which is due to their migratory lifestyle and their many alternative host plants.^24^

Globally, at least 50 parasitoid species have been reported to attack *M. persicae*,^22^ as listed by van Emden et al.^25^ In Australia, although many of these natural enemies are present, little research has been undertaken to investigate parasitoid species composition, the use of parasitoids as biological control agents of *M. persicae*, the thresholds required to suppress *M. persicae* populations, and the effect of seasonal changes on naturally occurring populations and parasitism rates. In one study on Australian grains, the proportion of mummies from total aphids rose from 1% to 15% as crop growth stage progressed, supporting other internationally observed trends.^23^

Assessing the future impact of a natural enemy is particularly important in predicting their contribution to preventing pest populations from reaching either an economic injury level or an economic threshold.^26^ This is of particular interest for aphid parasitoids, that can regulate pest populations when host densities are low.^27^ Furthermore, knowledge on aphid parasitism can help in timing chemical treatments to avoid periods when aphid parasitoids are present in large enough numbers to control aphids. These are important considerations in developing an alternative approach for aphid control particularly for *M. persicae* which is known to be resistant to over 80 insecticide active ingredients.^28^

Here, we considered several questions to provide an understanding of aphid parasitoid activity in *M. persicae* in canola fields. What are the general trends in *M. persicae*, alate, mummy, and aphid parasitoid numbers in Australian fields across a growing season? Based on previous studies,^23^ we expected mummification rates and parasitoid numbers to increase as the season progresses. Additionally, is the field OPR (estimated from in‐field mummy counts) of *M. persicae* representative of the laboratory OPR (laboratory‐reared parasitoid counts)? To investigate the causes of variability between field OPR and laboratory OPR, we considered parasitism rates: (i) across regions with different climates, (ii) across different scales, (iii) throughout the growing season, (iv) in the presence/absence of plant stress, (v) spatially across a field, in relation to the distance from the field edge, and (vi) within the context of aphid parasitoid composition (both primary and secondary). We hypothesized that differences between field OPR and laboratory OPR may increase under favorable climatic conditions for parasitoid development, as parasitoids would spend less time in the mummy stage, but we had no clear hypotheses about scale or plant stress. Due to the proximity to neighboring floral resources, we expected parasitism (and predation) may be higher at field edges. Finally, we expected estimates of field OPR and laboratory OPR to be affected by parasitoid composition. The reasoning being that secondary parasitism reduces the success of primary parasitism;^29^ therefore, if secondary parasitoids are present, their slower developmental time would increase the likelihood of observing mummies in the field.

## MATERIALS AND METHODS

### Site selection

**Figure 1:**
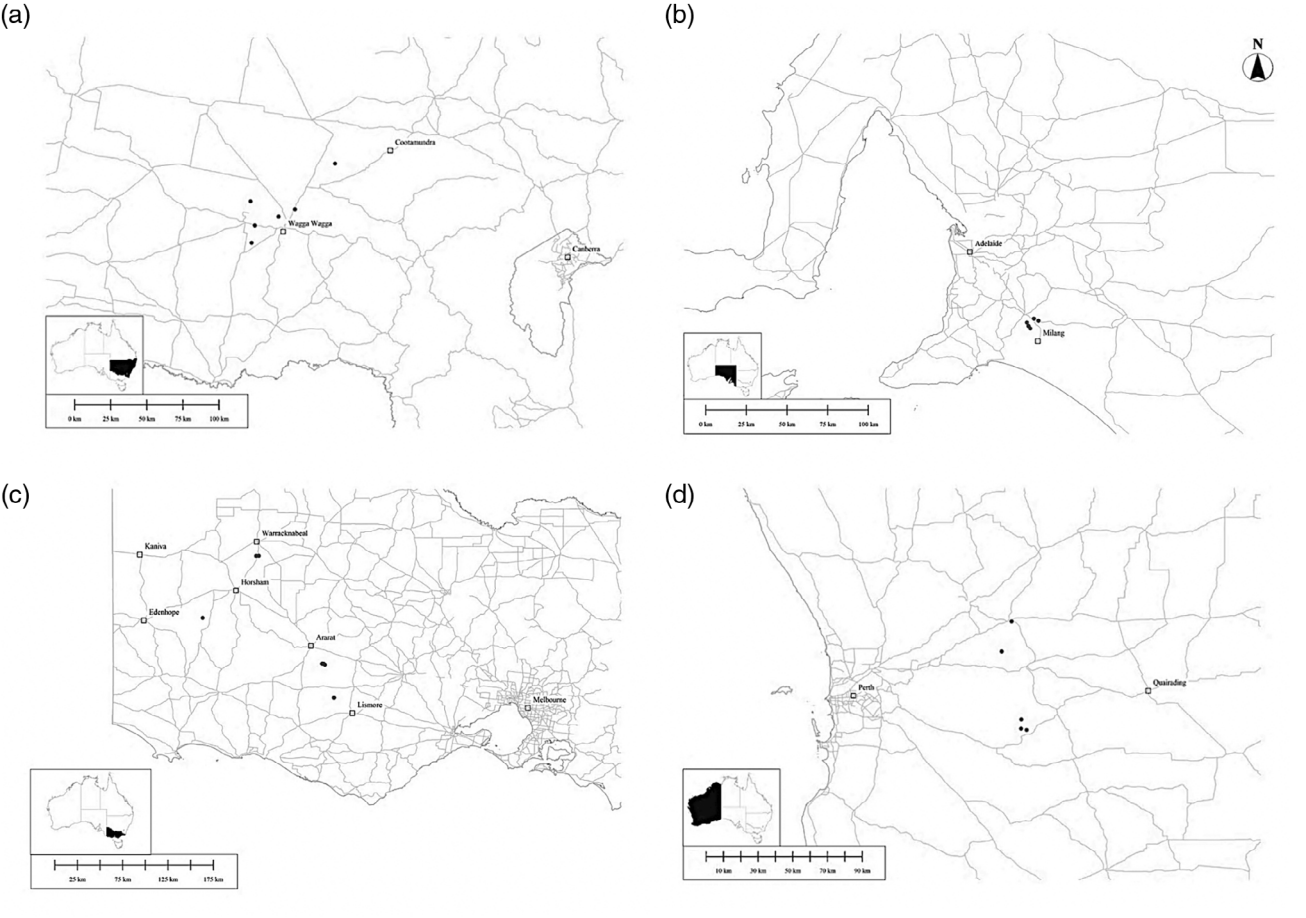
Map of field sites (shown as black circles) in each state: (a) NSW, (b) SA, (c) VIC, and (d) WA (inset maps depict states on Australian map).^30^, ^31^

In 2019, canola fields, at least 1 km from one another, were surveyed for *M. persicae* across the grain belt in each of the following states: NSW, South Australia (SA), Victoria (VIC), and Western Australia (WA). Between 10 and 13 fields were surveyed in each state, with every crop sown from canola seed coated with an insecticide treatment (as is standard practice in Australia). Selection of each field was based on minimal insecticide usage, with preference given to those fields where no chemical sprays were likely to be applied. Site visits were conducted from June/July 2019 until aphids were detected, although in several fields, aphids did not appear. The fields in which aphids were detected continued to be sampled every 4 weeks until the end of the season (as defined by the time of windrowing of the crop) (Fig. 1). There were five fields with *M. persicae* in SA and WA, six in NSW, and seven in VIC. Across the states and time points, there were 61 sampling trips undertaken in total. During the sampling period, VIC was generally colder and experienced more rainfall than the other states, with WA on average being warmer and drier.

### Field aphid collections

Field‐derived and laboratory‐derived data were electronically recorded using a mobile software application developed for this project by Andy Hulthen based on the Open Data Kit described in Hartung et al.^32^ The application accessed the in‐built GPS and location‐based services of the mobile phone as well as a barcode‐reading capability for recording individual labelled samples. The data were uploaded directly to a database in the Cloud (when in mobile internet range). Collectors followed prompts on the mobile application, noting several variables in the field, such as crop growth stage, plant condition, alates/non‐alates, presence of instars, and natural enemy presence (see Tables S1 and S2).

Canola plants within fields were sampled directly by hand for the presence of aphids and mummies, at sampling points at least 30 m from one another. Plant condition was categorized as ‘unstressed’, where plants looked healthy and turgid, or ‘stressed’ where plants were wilting, had a yellowing of or dull color to leaves, were stunted, patchy, had white feeding marks on the leaves, or a combination of the above. Stressed plants were targeted, and searches began at the edge of the crop, moving into the field, in a zigzag formation, reaching >100 m from the field edge. Plants at each sampling point were searched with a focus on the underside of the lower leaves of canola plants, the most common location for *M. persicae*.^33^ *Myzus persicae* was targeted and prioritized over other aphid species when present, although the presence of cabbage aphid (*Brevicoryne brassicae* L.) and turnip aphid (*Lipaphis erysimi* (Kaltenbach)), was noted.

Sampling points were inspected until eight points positive for *M. persicae* were logged. If *M. persicae* was absent at a sampling point, the location was recorded, and the next point sampled. Up to 24 sampling points were inspected at each field. Each sampling point was searched haphazardly for *M. persicae* aphids and mummies for 1 minute, or until the combined count of these reached 50 individuals, whichever occurred first. The count was capped due to constraints on time available to process the aphids. On a few occasions the sum of collected aphids and/or mummies exceeded 50. Aphids and mummies were kept on their respective leaves and stored, along with paper toweling and leaves with no aphids, in a sealed container. Each sampling container was labelled with a barcode that corresponded with the phone application record. Samples were kept cool during transport to the laboratory.

### Rearing of parasitoids

Parasitoids were reared from both aphids and mummies collected from the field and identified to species level, as described below. For material collected from NSW fields, data were not recorded on whether a parasitoid emerged from the mummies, or the aphids collected from the field. For the other states, these two groups were recorded separately. Consequently, NSW data were omitted from some analyses

### Rearing from field collected mummies

In the laboratory, all mummies collected from a sampling point were placed into a petri dish lined with moist filter paper. The underside of each petri dish was labelled with the corresponding barcode and placed within a controlled temperature (CT) cabinet maintained at 20 °C and a 16 L:8D photoperiod. For 2 weeks, Petri dishes were checked every second day for parasitoid emergence. Once a parasitoid emerged, it was stored in 80% ethanol, and labelled with a corresponding barcode. Parasitoids reared from the same sampling point were stored together. We refer to these field collected mummies that successfully reared parasitoids as ‘field mummies.’

### Rearing from field collected (non‐mummified) aphids

Petri dishes were made up with a 1% agar solution, within which 2–3 cotyledons of canola or sprouting radish (*Raphanus raphanistrum* subsp. *Sativus* L.) were inserted. The lid was lined with filter paper. Aphids from the same sampling point were placed onto the cotyledons, unless numbers were very high (~50 or above), in which case multiple petri dishes were used. Each dish was labelled on the underside with the corresponding barcode and placed within a CT cabinet, set as above. Leaves were changed weekly or as needed if limp or discolored, or if fungus began to appear. Any mummies that developed were removed and placed in a separate petri dish. These mummies were checked every second day for parasitoid emergence for 2 weeks from the date of collection. Once a parasitoid emerged, it was stored in 80% ethanol separate to the mummy case. We refer to these field collected aphids that reared parasitoids as ‘laboratory mummies.’

At the end of the 2 weeks, all aphids that remained unparasitized were stored in 80% ethanol. For NSW, parasitoids produced from field mummies and laboratory mummies were combined within the same tubes along with 80% ethanol. All parasitoids reared from *M. persicae* were stored at 4 °C and identified morphologically to species level, using keys by Rakhshani et al.^34^, ^35^

### Parasitoid identification using molecular approaches

For any parasitoid that required further clarification on species identification, we undertook CO1 barcoding. This included males with unclear morphological taxonomy and poorly preserved specimens. Parasitoid DNA was extracted non‐destructively using a modified Chelex® extraction method, adapted from Walsh et al,^36^ as detailed in Carew et al.^37^ An individual parasitoid was placed within a micro‐centrifuge tube, along with 3 μL of Proteinase K (20 mg/mL) and 70 μL of 5% Chelex® solution, before being incubated in a water bath. PCRs were undertaken using a 10% dilution of the DNA extractions, amplifying the samples with the ‘universal’ arthropod primer pair LCO1490/HCO2198.^38^ Reactions contained a final concentration of 1x Standard Taq Reaction Buffer (New England Biolabs, Massachusetts, USA), 2.5 mM MgCl_2_, 0.5 μM each primer, 0.2 mM dNTPs, 2.4 U IMMOLASE DNA Polymerase (Bioline, London, UK) and 3 μL diluted DNA, in a reaction volume of 30 μL. Amplicons were sent to the Australian Genome Research Facility for sequencing, before forward and reverse sequences were assembled and trimmed using Geneious version 9.1.8 (https://www.geneious.com). Sequences were identified using the Genbank database (http://www.ncbi.nlm.nih.gov) and cross‐referenced with the Barcode of Life Data System database (BOLD; http://www.barcodinglife.org).^39^ In 96% of samples, we were able to confidently assign a parasitoid identification by directly comparing our CO1 sequences to those published on Genbank and/or BOLD.

### Data analyses

Parasitism rates were calculated in two ways. The first was the ‘field observed parasitism’ rate (field OPR), the number of field mummies divided by the total number of aphids and mummies collected. The second was the ‘laboratory observed parasitism’ rate (laboratory OPR), defined as the total number of parasitoids that were reared divided by the number of aphids and mummies that were collected in the field. The ratio of parasitoids reared from mummies collected from the field (‘field mummies’) over the number reared from aphids collected from the field that subsequently became mummified (‘laboratory mummies’) was also investigated to assess the extent to which field mummy counts might underestimate parasitism levels. As most parasitoids reared belonged to one species (*Diaeretiella rapae* (M'Intosh) *–* see below) we considered overall parasitism rates rather than parasitism rates by individual species.

General Linear Mixed Models (GLMMs), with crop field as a random factor nested within states, were undertaken to analyze the effects of state, field, crop growth stage, and crop stress on the variables. Count data were log transformed (ln(X + 1)) prior to analysis as they were not normally distributed, while proportion data were logit transformed as recommended by Warton et al.^40^ We used this approach rather than treating the alates *versus* non‐alates, mummies *versus* non‐mummified aphids and reared parasitoids *versus* unparasitized aphids as binomial variables because of the uneven sampling and patchy distribution of aphids, and potentially parasitism, across fields. Note that proportions were based on the presence of at least 18 aphids/alates/mummies. Crop growth stage was considered a factor due to the categorical nature of the variable, and stages prior to flowering were excluded from analysis due to the rarity of aphids. *Post hoc* Tukey's multiple comparison tests were undertaken to determine which means were statistically different.

To explore the relationships between abundance of *M. persicae*, proportion of mummies from total aphids sampled, and proportion of parasitoids reared *versus* distance from field edges, GLMMs were performed on data with distance from edge as a covariate, after collating data across all crop growth stages. For these analyses, any fields with fewer than 10 aphids or parasitoids reared were removed.

For the analysis of parasitoid species composition, parasitoid counts were summed across sampling points within a field before multiple response permutation procedures (MRPPs) were used to investigate the effects of state, crop stress, crop growth stage, and field on parasitoid species composition, with Euclidean distance as a similarity measure. Paired t‐tests were undertaken to compare the proportion of *D. rapae* with other parasitoids when reared from laboratory mummies *versus* field mummies.

All analyses were conducted using Minitab version 19.1.0.0^41^ or IBM Statistics SPSS 24, with the exception of the MRPPs, which were performed in R version 4.0.1,^42^ using Rstudio version 1.3.959.^43^

## RESULTS

### General trends in aphid, alate, mummy, and aphid parasitoid numbers in the field

**Figure 2:**
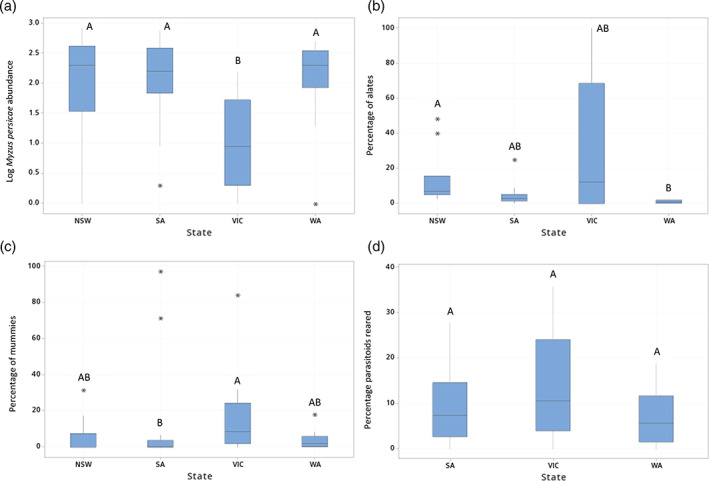
Box plots showing by state (a) *M. persicae* abundance per sampling point, and the percentage of (b) alates, (c) mummies, and (d) reared parasitoids from fields excluding those in NSW. [Outliers are indicated by single asterisks. Different letters indicate significant differences between states in *post hoc* tests].

During 2019, 11246 non‐mummified *M. persicae* were collected, with 3578 from sampling points in NSW, 4218 in SA, 585 in VIC, and 2865 in WA. In all states, *M. persicae* presence varied over time, with very few aphids found during the vegetative stage. In NSW, most sampling was undertaken during the flowering crop growth stage (43% of visits), in VIC during the flowering/podding stage (44%), in WA during the flowering/podding and podding/senescing stages (both 36%), and in SA during the podding/senescing crop growth stage (41%). Aphids were first recorded in low numbers in all states in mid‐July, except VIC in early August. In VIC, there were significantly fewer non‐mummified *M. persicae* per field than in the other states (Fig. 2(a)). There was no difference in aphid numbers collected per field at the three later crop growth stages (flowering, flowering/podding, podding/senescence) (Table 1). The proportion of alates from total aphids sampled was significantly higher in NSW than in WA (Table 1; Fig. 2(b)). Crop stress was found to have no effect on *M. persicae* abundance or the proportion of alates sampled (Table 1).

**Table 1:** Results of GLMMs testing the effect of state, field, crop growth stage and crop stress on *M. persicae* numbers, and proportions (logit transformed) of alates, mummies, and reared parasitoids considered at the field level

Organism and measurement	Factor	MS	F_(df1,df2)_	*P* value
Mean *M. persicae* abundance in the field	State	5.811	11.64_(3,19)_	**<0.001**
	Field (nested within state)	0.563	1.13_(19,35)_	0.369
	Crop growth stage	1.180	2.36_(2,35)_	0.109
	Crop stress	0.864	1.73_(1,35)_	0.197
	Error	0.499		
Proportion of alates (logits) in the field	State	5.158	4.84_(3,17)_	**0.009**
	Field (nested within state)	0.583	0.55_(17,25)_	0.899
	Crop growth stage	1.545	1.45_(2,25)_	0.253
	Crop stress	1.177	1.11_(1,25)_	0.303
	Error	1.065		
Proportion of mummies (logits) in the field	State	5.599	3.85_(3,19)_	**0.019**
	Field (nested within state)	2.311	1.59_(19,30)_	0.125
	Crop growth stage	41.643	28.61_(2,30)_	**<0.001**
	Crop stress	0.003	0.00_(1,30)_	0.962
	Error	1.456		
Proportion of reared parasitoids (exc. NSW) (logits) in total	State	2.088	1.78_(2,14)_	0.192
	Field (nested within state)	1.212	1.03_(14,23)_	0.459
	Crop growth stage	6.461	5.50_(2,23)_	**0.011**
	Crop stress	4.903	4.17_(1,23)_	0.053
	Error	1.175		

Figures shown in bold are statistically significant.

**Figure 3:**
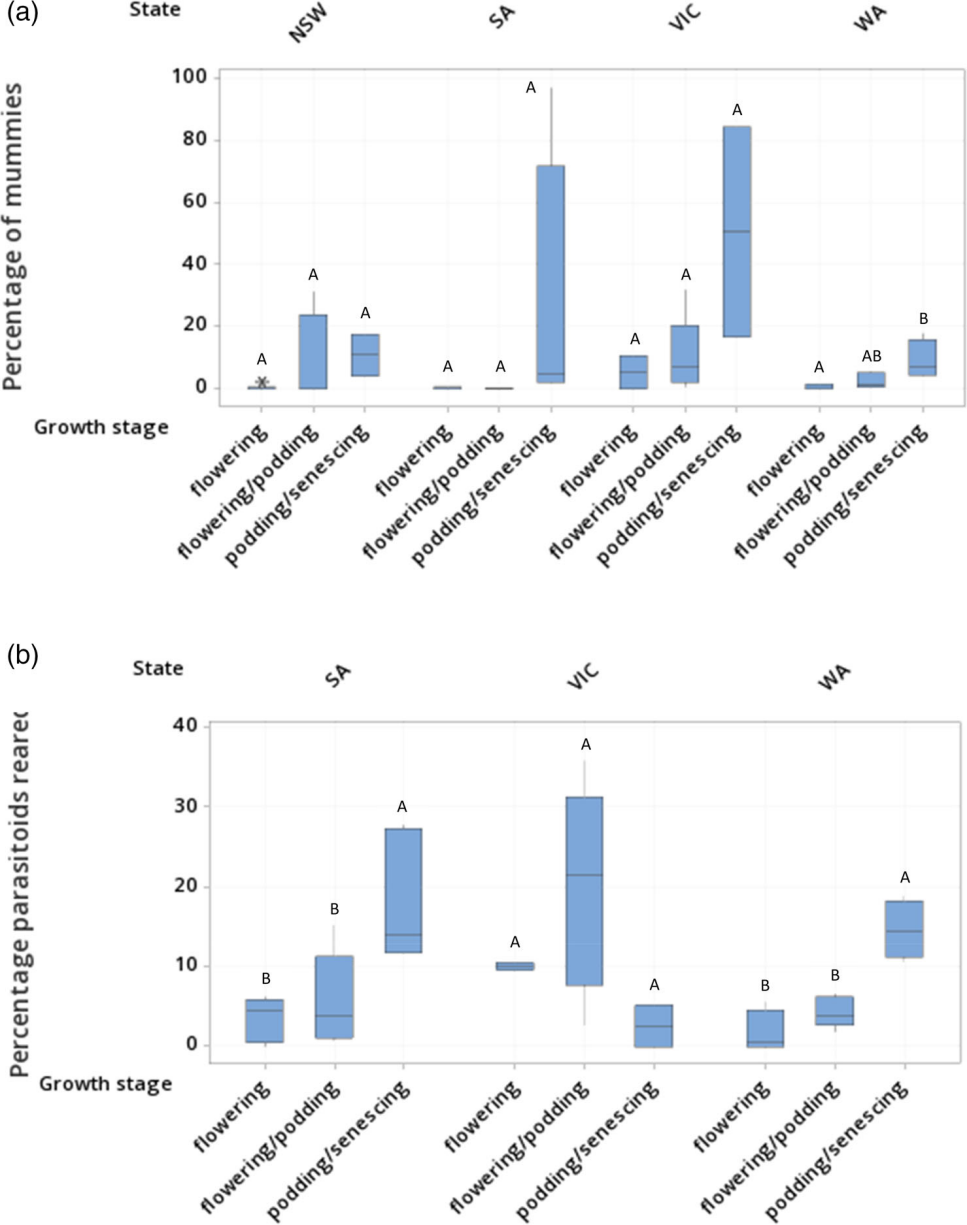
Box plots displaying effects of crop growth stage on (a) the percentage of samples collected as mummies per state, and (b) the percentage of samples collected which reared parasitoids per state. [Outliers are indicated by single asterisks. A *post hoc* test across each state found the percentage of mummies from total aphids collected was significantly higher during the podding/senescing crop growth stage than during flowering or flowering/podding. A *post hoc* test across each state found the percentage of mummies from total aphids collected was significantly higher during the podding/senescing crop growth stage than during flowering].

During 2019, 515 mummies were collected, with 145 in NSW (4% of total *M. persicae* collected in this state), 148 in SA (4%), 52 in VIC (9%) and 170 in WA (6%). Mummies were not found in NSW, SA, and WA until August, and until September in VIC. There were significantly higher proportions of mummies found in VIC than in SA (Table 1; Fig. 2(c)). A significantly higher proportion of mummies were collected during the podding/senescing stage than during the flowering and flowering/podding stages (Table 1; Fig. 3(a)). Only one mummy was found during the vegetative stage (from NSW). The increase in the proportion of mummies at later crop growth stages reflects an increase in field parasitism estimates consistent across all states, except NSW (Fig. 3(a)). The proportion of mummies from total aphids collected was not affected by field or by crop stress (Table 1).

Parasitism as measured by the total number of reared parasitoids showed a significant effect of crop growth stage, but no effect of state, field, or crop stress (Table 1). The proportion of reared parasitoids from total aphids and mummies collected increased with crop growth stage in both SA and WA, but not in VIC (Fig. 3(b)).

### Variability of field observed, and laboratory observed rates of parasitism

#### Variability across regions, different scales, throughout the growing season, and with plant stress

**Figure 4:**
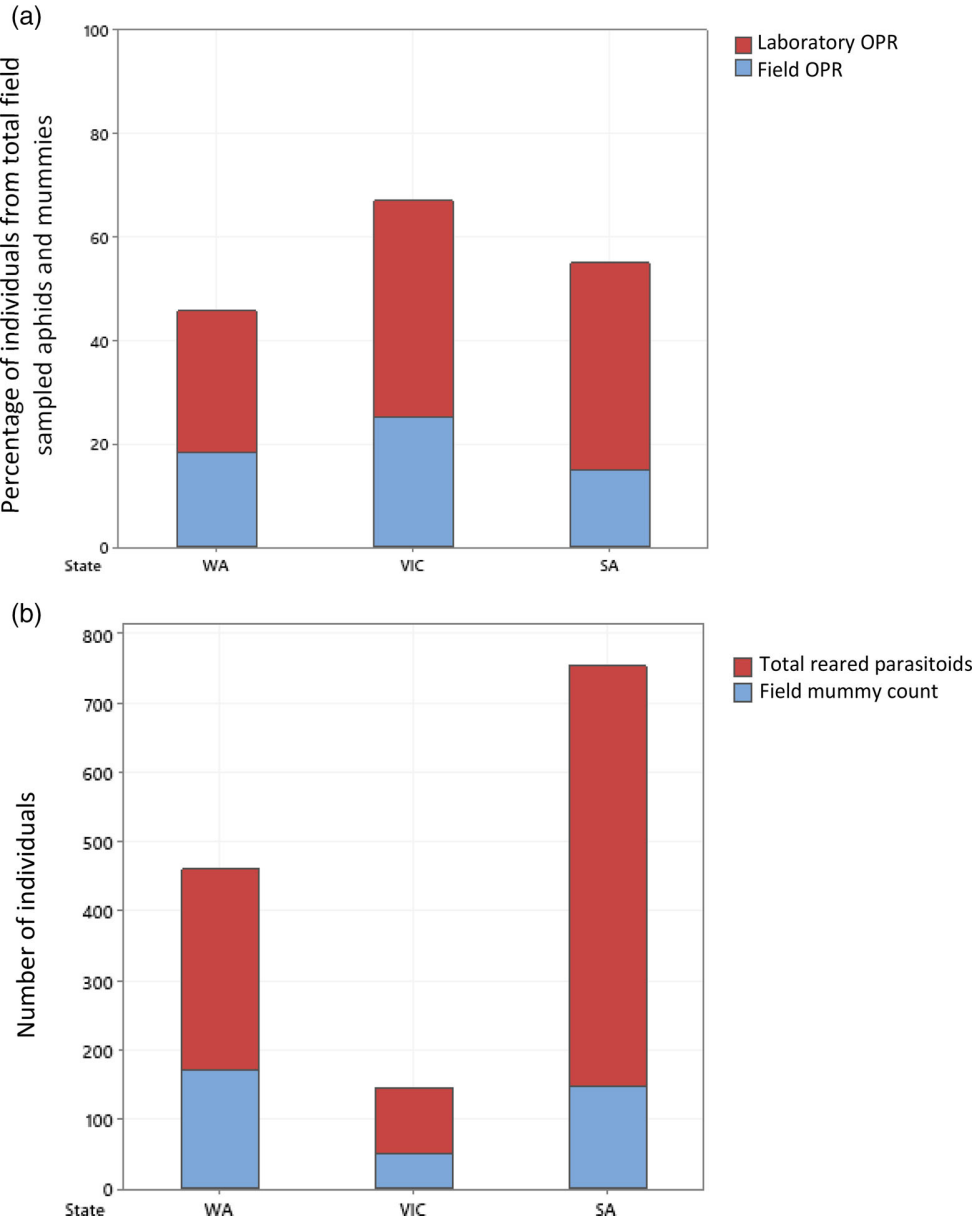
Stacked bar charts displaying for each state, excluding New South Wales (NSW): (a) laboratory observed parasitism rates [laboratory observed parasitism rate (OPR)], as the percentage of total parasitoids reared from total field samples aphids and mummies, and field observed parasitism rates (field OPR), as the percentage of total mummies collected from field sampled aphids and mummies, and (b) raw numbers of total reared parasitoids and field mummy counts.

At a state level, total parasitoids reared were always higher than total field mummies sampled (Fig. 4; Table 2). Whether parasitism occurred or not, laboratory OPR was higher than field OPR (Table 3; Fig. 4). However, 16.3% of fields had a lower laboratory OPR than field OPR due to rearing failures (Table 3). At a sampling point level within a field, 70.0% of sampling points had higher laboratory OPR than field OPR (Table 3). On a field level, when parasitism occurred, the difference in field OPR and laboratory OPR did not vary across state, field, crop growth stages or crop stress (GLMM: state MS = 0.364, F_2,12_ = 0.97; *P* = 0.397; field MS = 0.050, F_12,18_ = 0.13; *P* = 1.000; crop growth stage MS = 0.234, F_2,18_ = 1.18, *P* = 0.546; crop stress MS = 0.060, F_1,18_ = 0.16, *P* = 0.695; error MS = 0.374).

**Table 2:** Comparison of field mummies sampled, and parasitoids reared at a nationwide and state level

Scale	Total field mummies sampled	Total parasitoids reared	Total parasitoids reared as percentage of field mummies sampled
Nationwide	515	1221	237%
NSW	145	233	161%
SA	148	606	409%
VIC	52	92	177%
WA	170	290	171%

**Table 3:** Comparisons of laboratory observed parasitism rate (OPR) and field OPR at a field and sampling point level (nationwide and for each state)

Scale	Laboratory OPR > Field OPR	Laboratory OPR = Field OPR	Laboratory OPR < Field OPR
Nationwide; field (43)	62.8% (27)	20.9% (9)	16.3% (7)
Nationwide; sampling point (120)	70.0% (84)	20.8% (25)	9.2% (11)
South Australia (SA); field (17)	76.5% (13)	11.8% (2)	11.8% (2)
SA; sampling point (40)	97.5% (39)	2.5% (1)	0.0% (0)
Victoria (VIC); field (13)	38.5% (5)	38.5% (5)	23.1% (3)
VIC; sampling point (40)	50.0% (20)	40.0% (16)	10.0% (4)
Western Australia (WA); field (13)	69.2% (9)	15.4% (2)	15.4% (2)
WA; sampling point (40)	62.5% (25)	20.0% (8)	17.5% (7)

[Figures in brackets show raw numbers across different time points at a field level and combined time points at a sampling point level; NSW excluded].

**Figure 5:**
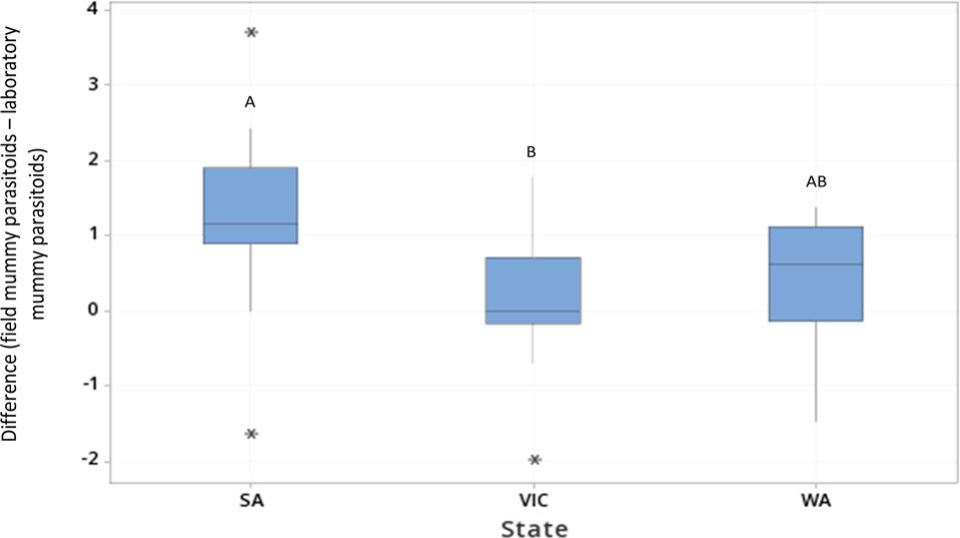
Box plot displaying effects of state on the difference between the number of parasitoids reared from laboratory mummies and the number of those reared from field mummies assessed at the field level. [Outliers are indicated by single asterisks. Different letters indicate significant differences between states in *post hoc* tests].

The field mummies, of which 370 were collected from SA, VIC, and WA, produced 280 parasitoids, with a successful rearing rate of 76%. Of the 7668 non‐mummified *M. persicae* observed in SA, VIC, and WA during this study, 708 (9%) became mummies and produced parasitoids within the laboratory (laboratory mummies). The aphids that became mummies but did not produce parasitoids were not categorized as ‘laboratory mummies’ or included in the analysis, as these have no bearing on the laboratory OPR. Of the parasitoids reared, those from laboratory mummies constituted 49% of total parasitoids reared in WA, 33% in VIC, and 83% in SA. The difference between the number of parasitoids reared from field mummies and those reared from laboratory mummies was not significantly different across field, crop growth stage, or crop stress, but was different across states (GLM: state MS = 8.275, F_2,14_ = 7.94, *P* = 0.002; field MS = 0.615, F_14,23_ = 0.59, *P* = 0.846; crop growth stage MS = 2.049, F_2,23_ = 1.97, *P* = 0.163; crop stress MS = 2.021, F_1,23_ = 1.94, *P* = 0.177; error MS = 1.042). At a field level, the variation between parasitoids reared from field mummies and laboratory mummies was significantly greater in SA than in VIC (Fig. 5).

#### Spatial variability within a field

Although our sampling in the field did not proceed perpendicularly from the field edge but proceeded in a zigzag fashion, the distance from the field edge varied suitably to consider distance effects within this context. Only the first eight sampling points (~210 m moved from the field edge) were used in the analysis as these sampling distances were repeated each visit, regardless of aphid presence/absence. Although there was a state and field effect on *M. persicae* abundance, there was no effect of distance from the field edge (GLMM: state MS = 61.436, F_3,19_ = 207.53; *P* < 0.001; field MS = 3.125, F_19,160_ = 10.56; *P* < 0.001; distance from field edge MS = 1.120, F_1,160_ = 3.78, *P* = 0.054; error MS = 0.296) (Fig. S3a). Again, although there was a state and field effect, the proportion of mummies sampled from *M. persicae* was not affected by distance from the field edge (GLMM: state MS = 3.087, F_3,19_ = 5.23; *P* = 0.002; field MS = 3.396, F_19,142_ = 5.75; *P* < 0.001; distance from field edge MS = 0.387, F_1,142_ = 0.65, *P* = 0.420; error MS = 0.591) (Fig. S3b). The proportion of parasitoids reared was also affected by state and field but not distance from the field edge (GLMM: state MS = 7.443, F_2,14_ = 8.69; *P* < 0.001; field MS = 3.647, F_14,99_ = 4.26; *P* < 0.001; distance from field edge MS = 0.295, F_1,99_ = 0.34, *P* = 0.558) (Fig. S3c). These data indicate a relatively consistent rate of mummification and parasitism across canola fields.

#### Variation with parasitoid community composition

**Figure 6:**
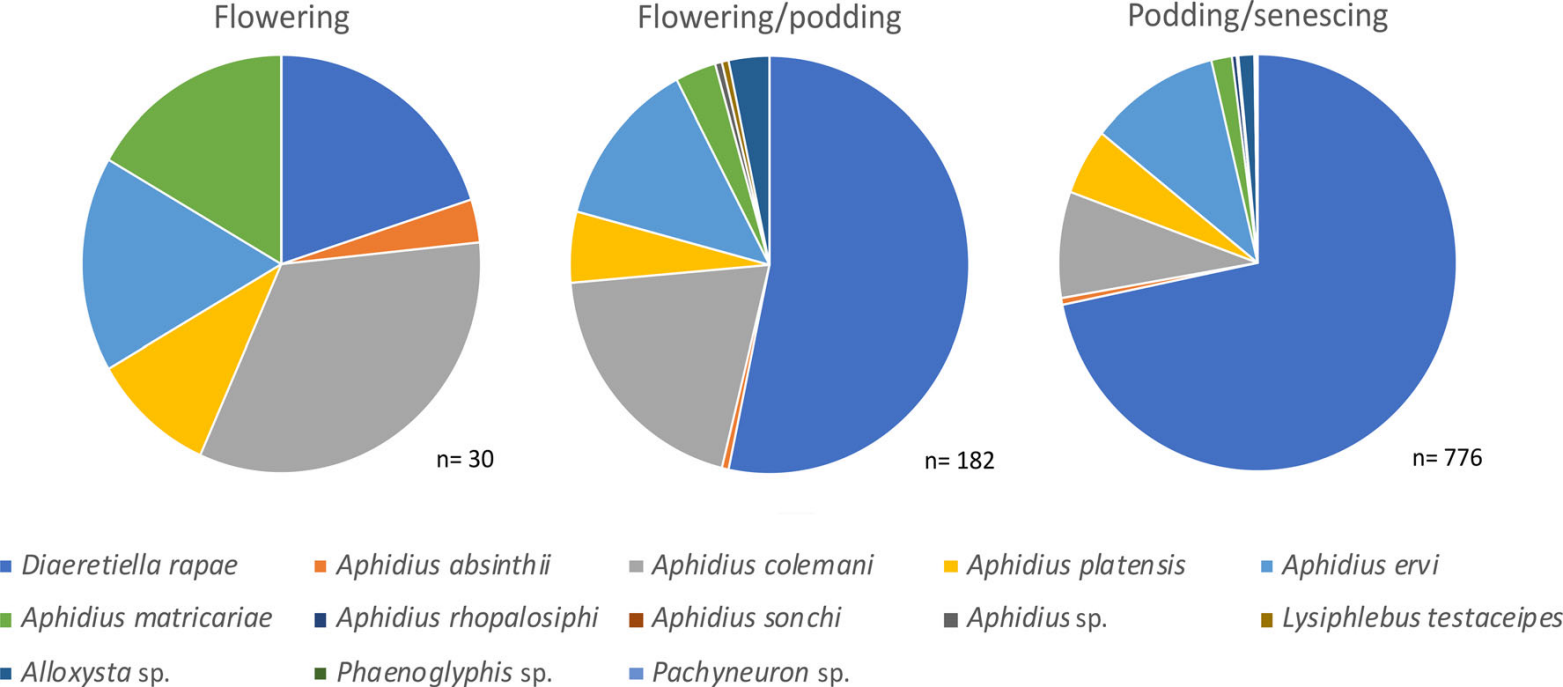
Parasitoid composition within canola fields during different crop growth stages. Note sample sizes below pie charts.

Primary parasitoids constituted approximately 98% of all parasitoids reared, hyperparasitoids approximately 2%, and mummy parasitoids 0.08%. Of the primary parasitoids, approximately 73% were *D. rapae*, approximately 10% *Aphidius ervi* Haliday, and approximately 9% *Aphidius colemani* Viereck, with the other species together constituting <8% of the total. No Aphelinidae emerged from the collected aphids. Further details of parasitoid composition by state are given in the supplementary material. Total parasitoid species composition was not different across fields (MRPP, A = 0.009, *P* = 0.293) or states (MRPP, A = 0.009, *P* = 0.264), nor did it differ with crop stress (MRPP, A = 0.009, *P* = 0.300). Crop growth stage, however, was found to have a significant effect on parasitoid species composition (MRPP, A = 0.239, *P* = 0.001), with a greater diversity of parasitoid species at later crop growth stages (Fig. 6).

## DISCUSSION

### General trends in aphid, alate, mummy, and aphid parasitoid numbers in the field

Aphids were first detected in canola fields in July in all states except VIC where the first detection occurred in August. Although possibly present in low numbers prior to this, we considered this the first successful colonization. The delayed establishment of aphids in the crop is most likely due to the efficacy of seed treatments containing systemic insecticides, which were applied at all sites, reducing over time. As expected, the first observation of mummies occurred 1 month after aphid colonizations. A delay between host and parasitoid emergence is generally observed, particularly in annual crops.^2^ Mummies were first collected in NSW, SA and WA in August, and September in VIC. Field parasitism was observed mostly between flowering and senescence, with parasitoids peaking during the flowering/podding and podding/senescing stages. Seasonal differences can cause populations of *M. persicae* and their parasitoids to vary between years.^23^ Reduced summer rainfall can result in fewer weeds and volunteer plant hosts persisting between cropping seasons. This restricted ‘green bridge’ provides limited refuge for beneficial insects and can delay infestation of host crops by insect pests such as aphids.^44^ With 2019 a drought‐affected year, further sampling should be undertaken across other seasons to further test this pattern.

*Myzus persicae* abundance and the proportion of alates were not affected by crop growth stage. This could be due to several overriding contributing factors that affect aphid population growth, such as high variability across sites, elevation, temperature, dew point,^45^ and the presence of natural enemies. Crop stress was not found to affect *M. persicae* abundance, or the proportions of alates, mummies, or parasitoids reared. There is a common assertion that the formation of aphids' wings (and in turn dispersal) is due to poor host‐plant nutritional quality. However other factors are known to play a role,^46^ such as overcrowding, which in turn causes deterioration of the host plant.^47^ Plants were identified as ‘stressed’ based on their appearance. This could have been caused by any number of factors, including but not limited to aphid damage, environment (i.e., moisture or heat stress), or other arthropods (i.e., mite feeding stress); many of these may not be relevant to aphids or their parasitoids.

Although other studies have found that as the season progresses parasitoids are more likely to die or diapause within developing mummies,^19^ the proportion of reared parasitoids increased as crop growth stage progressed in both SA and WA. The same was not found in VIC, which could be due to the later arrival of aphids and the subsequent lower proportion of mummies collected. This knock‐on effect could be attributed to more fields being sampled during the flowering growth stage and fewer during the podding/senescing stage than the other states. The proportion of reared parasitoids in VIC was highest during the flowering/podding stage. The flowering/podding crop growth stage in SA and WA spanned the months of August and September. However, in VIC some of the sampled crops were still flowering/podding in November (coinciding with podding/senescing in the other states). November temperatures in Australia are considerably higher than earlier in the growing season. For *A. colemani* and *Aphidius matricariae* Haliday, the time taken for mummies to form has been shown to be inversely correlated with temperature in the range of 10–25 °C.^48^ This suggests that in Australia, November temperatures are more suited for aphidiine development. Therefore, it would appear that the increase in proportion of reared parasitoids is due to temperature and seasonal differences more than crop growth stage itself.

### Variability of field observed, and laboratory observed rates of parasitism

Estimates of parasitism levels based on mummy counts or proportions of mummies (field OPR) only relate to numbers of parasitoids at pre‐pupal and pupal developmental stages.^19^ Around 9% of aphids in the field became mummies and successfully reared parasitoids in the laboratory (laboratory mummies). It must be noted, however, that rearing parasitoids within a laboratory protects them against factors that could reduce their emergence under field conditions.^49^ Parasitoids reared in the laboratory are protected from pathogens and predators, such as ladybirds, syrphids and lacewings, which can predate parasitized aphids.^50^ Some predators may prefer young, parasitized aphids over their unparasitized counterparts.^51^ Additionally, primary parasitoids developing from laboratory mummies are protected from secondary parasitoids (hyperparasitoids and mummy parasitoids). The few hyperparasitoids reared from field mummies may have subsequently parasitized other mummies under field conditions, thereby increasing hyperparasitism rates. To investigate how intraguild predation affects the measurement of parasitism rate, it would be worthwhile directly assessing predation of parasitized aphids in the field.

#### Variability across regions, different scales, throughout the growing season, and in the presence of plant stress

At the field level, reared parasitoid counts were 2.4 times higher than field mummy counts. The discrepancy was highest in SA, at 4.1 times greater. At the field level, laboratory OPR was higher than field OPR for 62.8% of the fields sampled. At the sampling point level, laboratory OPR was higher than field OPR in 70.0% of cases. These results suggest that at both scales, field OPR usually provides an underestimate of laboratory OPR. Due to differences among fields, it is difficult to identify sources of variation between these rates, but factors such as landscape composition, configuration, and complexity, as well as the availability of alternative hosts may be involved as noted in other studies.^52^, ^53^

The significantly greater difference between the number of parasitoids reared from field mummies and laboratory mummies per field in SA compared with other states could be due to sampling differences. More sampling occurred during the podding/senescing crop growth stage in SA (at 41%) compared with the other crop growth stages. In VIC, the least amount of sampling was undertaken during the podding/senescing stage. This was because unfavorable (extremely dry) conditions resulted in slower canola development. This could also explain the lower aphid counts in VIC, as aphids are attracted to the yellow colour of flowering canola^25^ and then tend to build up in numbers. Variation in laboratory OPR and field OPR might therefore be affected by crop growth stage. Other factors that may have a direct or indirect impact on aphid or parasitoid development include other arthropods creating competition among aphids, or micro‐environments differing amongst the fields sampled. Further investigation is warranted to identify the cause of the difference across the states in variation between field OPR and laboratory OPR.

#### Spatial variability within a field

Edge effects are common for some aphid species, with *B. brassicae* rarely detected further than 30 m into the fields studied,^54^ however we saw no evidence of this in *M. persicae* in the study here, which has also been observed in earlier surveys of canola aphids in WA (Severtson D, unpublished data). Field edges can enhance natural enemy presence in addition to pests. This could maintain an equilibrium in aphid numbers, as natural‐enemy mediated edge effects have been documented within some agro‐ecosystems.^55^

#### Variation with parasitoid community composition

The parasitoid community was mostly comprised of primary parasitoids, with *D. rapae* the dominant species. Parasitoid species composition was not affected by state, field, or crop stress, but was affected by crop growth stage, with *D. rapae* becoming more dominant as crop growth stage progressed. This could reflect warmer temperatures during the latter growth stages, as *D. rapae* developmental time decreases more rapidly than that of other aphidiines, such as *A. matricariae*, as temperature increases.^56^ *Diaeretiella rapae* appears very tolerant to drought (in the absence of increased temperature), with similar or even higher rates of aphid parasitism under dry conditions.^57^ This could explain the dominance of this species in southern Australia in this study given 2019 was a drought‐affected year. Additionally, *D. rapae* has a very broad range of host aphids and plants compared with other aphidiines, and so can host swap and build up populations outside of the growing season.^58^ More *D. rapae* were reared in WA. This was likely due to the higher abundance of *B. brassicae* observed in this state, which is reputedly the preferred host of *D. rapae*.^59^ *Diaeretiella rapae* may not respond to chemical attractants from aphids, as increasing aphid densities did not affect the arrival time of this parasitoid.^60^ Instead, the parasitoid may respond to volatile compounds from cruciferous crops.^61^, ^62^, ^63^ *Diaeretiella rapae* orientates towards mustard oils produced by crucifers, causing this species of parasitoid to commonly attack aphids on cruciferous crops.^64^

It is important to note that our analyses of parasitism rates were undertaken combining data from all parasitoid species reared. Species apart from *D. rapae* were reared in low numbers. It would be valuable to investigate the relationship between the field OPR and laboratory OPR for each parasitoid species in areas where sufficient numbers can be collected. With growers unlikely to identify individual species, our focus here was on estimates that could be linked to data likely to be collected in a field. Secondary parasitoids, which include hyperparasitoids (those attacking the living aphid with delayed development) and mummy parasitoids (those attacking the mummified aphid with instantaneous development), were also very low in abundance throughout this study. Accurately assessing the presence of hyperparasitoids and mummy parasitoids is important given they can affect the long‐term ability of primary parasitoids to control *M. persicae* populations.^65^, ^66^ The difference between field OPR and laboratory OPR varied little between primary and secondary parasitoids, and between *D. rapae* and other primary parasitoids. Consequently, parasitoid species composition had no discernible effect on field OPR and laboratory OPR in our study region.

## CONCLUSION

Field OPR and laboratory OPR of *M. persicae* can vary considerably, due to a number of variables ranging from climatic factors to faunal composition. Parasitoid species composition, however, appeared to have no effect on the difference between field OPR and laboratory OPR. The number of parasitoids reared on average was over double the number of mummies sampled, and over four times higher in the state of SA. Furthermore, field OPR was greater than laboratory OPR at 62.8% of fields and 70.0% of sampling points surveyed. Consequently, mummy counts and field OPR do not provide a clear representation of laboratory OPR, generally providing an underestimate and varying across geographic regions and within the growing season. This information can be incorporated into decision making by agronomists and growers when determining thresholds for spraying aphids.

## CONFLICT OF INTEREST DECLARATION

The authors declare no conflict of interest.

## Supporting information


**Table S1:** Field data acquired using the mobile software application.
**Table S2:** Laboratory data acquired using the mobile software application.
**Fig. S1**: Association of reared parasitoid numbers with laboratory mummy counts during the (a) flowering/podding stage, and (b) podding/senescing stage [counts as (ln(x + 1))].
**Fig. S2**: Association of reared parasitoid numbers with field mummy counts during the (a) flowering/podding stage, and (b) podding/senescing stage [counts as (ln(x + 1))].
**Fig. S3:** Effect of distance from paddock edge on (a) logged *M. persicae* abundance (ln(x + 1)), percentage of (b) mummies, and (c) percentage of reared parasitoids.
**Fig. S4:** Species composition of parasitoids reared from *M. persicae* from Australian canola fields in this study.

## Data Availability

The data that support the findings of this study are available from the corresponding author upon reasonable request.

## References

[1] Powell W , Walton MP and Jervis MA , Populations and communities, in Insect Natural Enemies, ed. by Jervis MA and Kidd N . Chapman & Hall, London, pp. 223–292 (1996).

[2] Godfray HCJ , Parasitoids, Behavioral and Evolutionary Ecology. Princeton University Press, Princeton, p. 488 (1994).

[3] Oakley JN , Walters KFA , Ellis SA , Green DB , Watling M and Young JEB , Development of selective aphicide treatments for integrated control of summer aphids in winter wheat. Ann Appl Biol 128:423–436 (1996).

[4] Bodlah I , Muhammad N and Ul Mohsin A , Distribution, hosts and biology of *Diaeretiella rapae* (M'Intosh) (Hymenoptera: Braconidae: Aphidiinae) in Punjab, Pakistan. Pak J Zool 44:1307–1315 (2012).

[5] Sigsgaard L , The temperature‐dependent duration of development and parasitism of three cereal aphid parasitoids, *Aphidius ervi, A. rhopalosiphi*, and *Praon volucre* . Entomol Exp Appl 95:173–184 (2003).

[6] Gillespie DR , Nasreen A , Moffat CE , Clarke P and Roitberg BD , Effects of simulated heat waves on an experimental community of pepper plants, green peach aphids and two parasitoid species. Oikos 121:149–159 (2012).

[7] Jones MG , Abundance of aphids on cereals from before 1973 to 1977. J Appl Ecol 16:1–22 (1979).

[8] Colfer RG and Rosenheim JA , Predation on immature parasitoids and its impact on aphid suppression. Oecologia 126:292–304 (2001). 28547629 10.1007/s004420000510

[9] Meyhöfer R and Hindayana D , Effects of intraguild predation on aphid parasitoid survival. Entomol Exp Appl 97:115–122 (2000).

[10] Calvo D and Fereres A , The performance of an aphid parasitoid is negatively affected by the presence of a circulative plant virus. BioControl 56:747–757 (2011).

[11] Ye Z , Vollhardt IMG , Parth N , Rubbmark O and Traugott M , Facultative bacterial endosymbionts shape parasitoid food webs in natural host populations: a correlative analysis. J Anim Ecol 87:1440–1451 (2018). 29928757 10.1111/1365-2656.12875 PMC6099228

[12] Chau A and Mackauer M , Preference of the aphid parasitoid *Monoctonus paulensis* (Hymenoptera: Braconidae, Aphidiinae) for different aphid species: female choice and offspring survival. BioControl 20:30–38 (2001).

[13] Perdikis DC , Lykouressis DP , Garantonakis NG and Iatrou SA , Instar preference and parasitization of *Aphis gossypii* and *Myzus persicae* (Hemiptera: Aphididae) by the parasitoid *Aphidius colemani* (Hymenoptera: Aphidiidae). Eur J Entomol 101:333–336 (2004).

[14] Aqueel MA , Raza AM , Balal RM , Shahid MA , Mustafa I , Javaid MM *et al*., Tritrophic interactions between parasitoids and cereal aphids are mediated by nitrogen fertilizer. Insect Sci 22:813–820 (2014). 24623663 10.1111/1744-7917.12123

[15] Longley M , A review of pesticide effects upon immature aphid parasitoids within mummified hosts. Int J Pest Manag 45:139–145 (1999).

[16] Cheng S , Lin R , Yu C , Sun R and Jiang H , Toxic effects of seven pesticides to aphid parasitoid, *Aphidius gifuensis* (Hymenoptera: Braconidae) after contact exposure. Crop Prot 145:105634 (2021).

[17] Lowery DT and Isman MB , Toxicity of neem to natural enemies of aphids. Phytoparasitica 23:297–306 (1995).

[18] Nielsen C and Hajek AE , Control of invasive soybean aphid, *Aphis glycines* (Hemiptera: Aphididae), populations by existing natural enemies in New York state, with emphasis on entomopathogenic fungi. Environ Entomol 34:1036–1047 (2005).

[19] Walton MP , Powell W , Loxdale HD and Allen‐Williams L , Electrophoresis as a tool for estimating levels of hymenopterous parasitism in field populations of the grain aphid, *Sitobion avenae* (F.) (Hemiptera: Aphididae). Entomologia Experimentalis Et Applicata 54:271–279 (1990).

[20] Giles KL , Jones BG , Royer TA , Elliott NC and Kindler SD , Development of a sampling plan in winter wheat that estimates cereal aphid parasitism levels and predicts population suppression. J Econ Entomol 96:975–982 (2003). 12852644 10.1603/0022-0493-96.3.975

[21] Zeck EH , The Green Peach Aphid, Myzus Persicae (Sulzer), Vol. 43. Agricultural Gazette, New South Wales, pp. 611–616 (1928).

[22] Waterhouse DF and Sands DPA , Classical Biological Control of Arthropods in Australia. CSIRO Publishing, Canberra (2001).

[23] Ward SE , Umina PA , Macfadyen S and Hoffmann AA , Hymenopteran parasitoids of aphid pests within Australian grain production landscapes. Insects 12:44 (2021). 33430084 10.3390/insects12010044 PMC7827963

[24] Ro TH , Analysis and Simulation of the Dynamics of a Myzus Persicae (Homoptera: Aphididae)‐Predator Complex Community in the Central Basin of Washington. PhD disseration. Washington State University, Washington (1995).

[25] Van Emden HF , Eastop VF , Hughes RD and Way MJ , Ecology of *Myzus persicae* . Annu Rev Entomol 14:197–270 (1969).

[26] Zadoks JC , On the conceptual basis of crop loss assessment: the threshold theory. Annu Rev Phytopathol 23:455–473 (1985).

[27] Leblanc A and Brodeur J , Estimating parasitoid impact on aphid populations in the field. Biological Control 119:33–42 (2018).

[28] Mota‐Sanchez D and Wise JC , The Arthropod Pesticide Resistance Database, Michigan State University. https://www.pesticideresistance.org/. (2022).

[29] Greathead O , Insect parasitoids, in Parasitoids and Classical Biological ontrol, ed. by Waage J. and Greathead O . Academic Press, London, pp. 289–318 (1986).

[30] Public Service Mapping Agency . 2020. Administrative boundaries. https://data.gov.au/dataset/ds‐dga‐bdcf5b09‐89bc‐47ec‐9281‐6b8e9ee147aa/details?q=PSMA_administrative_boundaries [May 28 2020]

[31] Department of Agriculture and Water Resources . 2020. MCAS‐S Data. http://mcas.ternlandscapes.net.au/mcas-s/data.html [May 28 2020]

[32] Hartung C , Lerer A , Anokwa Y , Tseng C , Brunette W and Borriello G . Open Data Kit: Tools to Build Information Services for Developing Regions. Proceedings of the 4^th^ ACM/IEEE International Conference on Information and Communication Technologies and Development (ICTD); December 13–16, 2010; London (2010).

[33] Sarwar M , Comparative suitability of soil and foliar applied insecticides against the aphid *Myzus persicae* (Sulzer) (Aphididae: Hemiptera) in canola *Brassica napus* L. Int J Sci Res Environ Sci 1:138–143 (2013).

[34] Rakhshani E , Kazemzadeh S , Starý P , Barahoei H , Kavallieratos NG , Ćetković A *et al*., Parasitoids (Hymenoptera: Braconidae: Aphidiinae) of northeastern Iran: Aphidiine‐aphid‐plant associations, key and description of a new species. J Insect Sci 12:143–126 (2012). 10.1673/031.012.14301 PMC3648336 23463939

[35] Rakhshani E , Starý P , Tomanović Z and Mifsud D , Aphidiinae (Hymenoptera, Braconidae) aphid parasitoids of Malta: review and key to species. Bull Entomol Soc Malta 7:121–137 (2015).

[36] Walsh PS , Metzger DA and Higuchi R , Chelex‐100 as a medium for simple extraction of DNA for PCR‐based typing from forensic material. Biotechniques 10:506–513 (1991). 1867860

[37] Carew ME , Pettigrove V and Hoffmann AA , Identifying chironomids (Diptera: Chironomidae) for biological monitoring with PCR‐RFLP. Bull Entomol Res 93:483–490 (2003). 14704094 10.1079/ber2003268

[38] Folmer O , Black M , Hoeh W , Lutz R and Vrijenhoek R , DNA primers for amplification of mitochondrial cytochrome c oxidase subunit I from diverse metazoan invertebrates. Mol Mar Biol Biotechnol 3:294–299 (1994). 7881515

[39] Ratnasingham S , Hebert PDN and Barcoding BOLD , The barcode of life data system (www.Barcodinglife.Org). Molecular Ecology Notes 7:355–364 (2007). 18784790 10.1111/j.1471-8286.2007.01678.x PMC1890991

[40] Warton DI and Hui FK , The arcsine is asinine: the analysis of proportions in ecology. Ecology 92:3–10 (2011). 21560670 10.1890/10-0340.1

[41] Minitab . 2019. Minitab 19 Statistical Software. http://www.minitab.com/ [Accessed November 28 2020]

[42] R Core Team , R: A Language and Environment for Statistical Computing. R Foundation for Statistical Computing, Vienna (2020).

[43] Rstudio Team , Rstudio: Integrated Development Environment for R. Rstudio, Inc, Boston (2020).

[44] Maino J , Pirtle E , van Helden M , Heddle T and Umina A . 2020. How does climate affect the green bridge (and consequently aphid risk)? https://grdc.com.au/resources-and-publications/grdc-update-papers/tab-content/grdc-update-papers/2020/02/how-does-climate-affect-the-green-bridge-and-consequently-aphid-risk [Accessed March 28 2021]

[45] Klein ML , Rondon SI , Walenta DL , Zeb Q and Murphy AF , Spatial and temporal dynamics of aphids (Hemiptera: Aphididae) in the Columbia Basin and northeastern Oregon. J Econ Entomol 110:1899–1910 (2017). 28510728 10.1093/jee/tox134

[46] Müller CB , Williams IS and Hardie J , The role of nutrition, crowding and interspecific interactions in the development of winged aphids. Ecol Entomol 26:330–340 (2001).

[47] Swenson KG , Role of aphids in the ecology of plant viruses. Annu Rev Phytopathol 6:351–374 (1968).

[48] Zamani AA , Talebi A , Fathipour Y and Baniameri V , Effect of temperature on life history of *Aphidius colemani* and *Aphidius matricariae* (Hymenoptera: Braconidae), two parasitoids of *Aphis gossypii* and *Myzus persicae* (Homoptera: Aphididae). Environ Entomol 36:263–271 (2007). 17445360 10.1603/0046-225X-36.2.263

[49] Hughes GE , Sterk G and Bale JS , Thermal biology and establishment potential in temperate climates of the aphid parasitoid, *Lysiphlebus testaceipes* . BioControl 56:19–33 (2011).

[50] Meyhöfer R and Klug T , Intraguild predation on the aphid parasitoid *Lysiphlebus fabarum* (Marshall) (Hymenoptera: Aphidiidae): mortality risks and behavioral decisions made under the threats of predation. Biological Control 25:239–248 (2002).

[51] Rocca M and Messelink GJ , Combining lacewings and parasitoids for biological control of foxglove aphids in sweet pepper. J Appl Entomol 141:402–410 (2017).

[52] Roschewitz I , Hücker M , Tscharntke T and Thies C , The influence of landscape context and farming practices on parasitism of cereal aphids. Agr Ecosyst Environ 108:218–227 (2005).

[53] Plećas M , Gagić V , Janković M , Petrović‐Obradović O , Kavallieratos NG , Tomanović Z *et al*., Landscape composition and configuration influence cereal aphid‐parasitoid‐hyperparasitoid interactions and biological control differentially across years. Agr Ecosyst Environ 183:1–10 (2014).

[54] Severtson D , Detection and Monitoring of Cabbage Aphids (Hemiptera: Aphididae) in Dryland Canola. Doctor of Philosophy, The University of Western Australia, Brassicaceae: Brassicales (2016).

[55] Rand TA , Tylanakis JM and Tscharntke T , Spillower edge effects: the dispersal of agriculturally subsidized insect natural enemies into adjacent natural habitats. Ecol Lett 9:603–614 (2006). 16643305 10.1111/j.1461-0248.2006.00911.x

[56] Bernal J and Gonzalez D , Temperature requirements of 4 parasites of the Russian wheat aphid *Diuraphis‐noxia* . Entomol Exp Appl 69:173–182 (1993).

[57] Romo CM and Tylanakis JM , Elevated temperature and drought interact to reduce parasitoid effectiveness in suppressing hosts. PLoS One 8:e58136 (2013). 23472147 10.1371/journal.pone.0058136 PMC3589357

[58] Žikić V , Lazarević M and Milošević D , Host range patterning of parasitoid wasps Aphidiinae (Hymenoptera: Braconidae). Zool Anz 268:75–83 (2017).

[59] Hafez M , Seasonal fluctuations of population density of the cabbage aphid, *Brevicoryne brassicae* (L.) in The Netherlands and the role of its parasite, *Aphidus (Diaeretiella) rapae* (Curtis). Tijdschrift Over Plantenziekten 67:445–548 (1961).

[60] Lester PJ and Holtzer TO , Patch and prey utilization behaviors by *Aphelinus albipodus* and *Diaeretiella rapae* (Hymenoptera: Aphelinidae and Aphidiidae) on Russian wheat aphid (Homoptera: Aphididae). Biological Control 24:183–191 (2002).

[61] Shukla AN , Singh R and Tripathi CPM , Effect of food plants on the functional response of an aphid parasitoid *Diaeretiella rapae* (McIntosh) (Hymenoptera:Aphidiidae). Phytophaga 4:53–60 (1992).

[62] Vaughn TT , Antolin MF and Bjostad LB , Behavioral and physiological responses of *Diaeretiella rapae* to semiochemicals. Entomol Exp Appl 78:187–196 (1996).

[63] Rehman A and Powell W , Host selection behaviour of aphid parasitoids (Aphidiidae: Hymenoptera). J Plant Breed Crop Sci 2:299–311 (2010).

[64] Read DP , Feeny PP and Root RB , Habitat selection by the aphid parasite *Diaeretiella rapae* (Hymenoptera: Braconidae) and hyperparasite *Charips brassicae* (Hymenoptera: Cynipidae). Canadian Entomol 102:1567–1578 (1970).

[65] Müller CB and Godfray HCJ , Indirect interactions in aphid‐parasitoid communities. Res Popul Ecol 41:93–106 (1999).

[66] Müller CB , Adriaanse CT , Belshaw R and Godfray HCJ , The structure of an aphid‐parasitoid community. J Anim Ecol 68:346–370 (1999).

